# Retraction Note: Production of recombinant *Entamoeba histolytica*pyruvate phosphate dikinase and its application in a lateral flow dipstick test for amoebic liver abscess

**DOI:** 10.1186/s12879-022-07708-5

**Published:** 2022-09-08

**Authors:** Syazwan Saidin, Muhammad Hafiznur Yunus, Nor Dyana Zakaria, Khairunisak Abdul Razak, Lim Boon Huat, Nurulhasanah Othman, Rahmah Noordin

**Affiliations:** 1 Institute for Research in Molecular Medicine, Universiti Sains Malaysia, 11800 Penang, Malaysia; 2 School of Materials and Mineral Resources Engineering, Engineering Campus, Universiti Sains Malaysia, Nibong Tebal, 14300 Penang, Malaysia; 3 NanoBiotechnology Research and Innovation (NanoBRI), Institute for Research in Molecular Medicine, Universiti Sains Malaysia, 11800 Penang, Malaysia; 4 School of Health Sciences, Universiti Sains Malaysia, Kubang Kerian, 16150 Kelantan, Malaysia

## Retraction Note to: BMC Infectious Diseases 2014, 14:182 https://doi.org/10.1186/1471-2334-14-182

The Editor has retracted this article. After publication, concerns were raised about unexpected similarities in Figs. 1 and 2. They were addressed in an Erratum, but additional concerns emerged, specifically:

- Apparent similarities of the backgrounds in lanes 4 and 5 in Fig. 2 in the original article, as well as lanes 3–4 in Image 1A in the correction [1].
- Apparent similarities of the backgrounds in lane 2 of Fig. 1 and lane 2 of Fig. 2 in the original article, as well as lanes 1 and 6 in Image 1A in the correction [1].

The authors have provided further explanation for the figure similarity, however the Editor found these to not be satisfactory. Furthermore, the authors were unable to provide evidence of the ethics approval for the study. None of the authors agree to this retraction.
